# The Story of the Insane from Year to Year

**Published:** 1896-09-19

**Authors:** 


### THE STORY OF THE INSANE FROM YEAR TO YEAR.

### THE LIMERICK DISTRICT ASYLUM.

The limits of accommodation are given at 500 patients, but 575 were in residence at the end of the year. There were 120 admissions, 46 recoveries, and 39 deaths. The recoveries stand at 38·3 per cent. of the admissions, and the deaths at 6·9 per cent. on the average number resident. Intemperance in drink is given as the cause of insanity in 8 of the admissions, domestic trouble in 3, and hereditary influence in 35. Cerebral and spinal diseases are responsible for only 6 of the deaths. Progressive paralysis is not present in the death-table, and it is partly owing to the rarity of this disease that the death-rate is so low in Irish asylums. On the other hand, consumption seems very common in Limerick, for no fewer than 15 died of that disease during the year. An education table is given, and we learn that of the 575 patients in the asylum 51 have been well educated, 153 can read and write well, while 76 can neither read nor write; 81 can read only, and 173 are indifferently educated as regards reading and writing. It would seem that the asylum is in urgent need of additional space; and, although many special reports have been made, nothing has been done to remedy the overcrowding which exists, and which is one of the worst evils a medical superintendent can be confronted with. When a board of management does not anticipate the requirements of the asylum, it almost certainly lags behind them; and this dilatoriness is not really economy. It is merely a postponement of the work which must be done—a postponement which has so many obvious inconveniences that it is not necessary to refer to them. The Commissioner in Lunacy connects the high death-rate from consumption with the overcrowding, and there can hardly be a doubt he is right. Dr. O'Neil continues his lectures to the staff, and 14 of the attendants have obtained the nursing certificate of the Medico-Psychological Association. The Commissioner says that bearing in mind the overcrowded state of the asylum, the general condition was most creditable to the medical superintendent. The average cost per head per annum was £21 16s.

### HEREFORD COUNTY ASYLUM.

The number of patients in this asylum increased from 386 to 393. There were 51 first admissions, 28 readmissions, 22 recoveries, 31 discharged relieved, and 17 deaths. The percentage of recoveries stands at 28·2, and the deaths at 6·5, both of these being below the average of county asylums. Of the deaths, 6 were owing to brain diseases, 1 to tuberculosis, and 3 to cancer. Hereditary tendency caused the insanity in 17 of the admissions, drink in 9, while "previous attacks" are down in 20 cases, and 21 were congenital. The asylum is practically full, and Dr. Chapman has tried to remedy this evil by transferring patients to the workhouses, but he found, what others have found before him, that most of the patients were returned to the asylum. There is, indeed, no particular wish on the part of guardians and workhouse officials to burden themselves with the care of the insane. There has been an upward tendency in the rates of maintenance of our county asylums during the last few years, and Dr. Chapman accurately attributes part of this to the Local Government Act of 1888 and the Lunacy Act of 1889. "The effect of recent legislation," he says, "has been to insist on a large increase of certificates, reports, signing and countersigning of orders, checking and counterchecking everything in an elaborate system of book-keeping, more time being taken in recording and certifying what done than there is time to do it in." And he adds that "detailed personal supervision is replaced by office work and red tape." Unfortunately, this is all too true, and it has been pointed out again and again; but not the slightest attempt has been made by the Legislature to amend it. The committee "express their satisfaction with Dr. Chapman's able superintendence and unremitting attention to all his duties." The weekly cost per head is 9s.

### BETHLEM HOSPITAL.

There were 229 patients in this asylum on January 1st, and the number had fallen to 217 by the last day of December. There were 213 first admissions, 53 re-admissions, 145 recoveries, 30 discharged relieved, and 20 deaths. The percentage of recoveries on admissions was 54·5, and that of the deaths was 8·8 on the average number resident. Of course, no comparison can be made between Bethlem Hospital and our county asylums, because the former admits chiefly recent and presumably curable cases, while the latter admit all sorts and conditions of patients. Intemperance in drink accounts for 22 of the year's admissions, hereditary influence for 116, and moral causes for 81. One incident occurred during the year which emphasises a serious blot in the new Lunacy Act. Patients have a right to obtain a personal interview with a justice. One patient exercised this right; the justice considered that he had recovered (although the medical officers did not), and the Lunacy Commissioners directed that he should be discharged. Within a very short time of discharge the man assaulted his wife, inflicting a wound on her head which might have had serious consequences, and the matter was put into the hands of the police. Had the woman died who would have borne the blame? Many of the attendants and nurses have received the certificate of the Medico-Psychological Association; but it does not appear that a more extended course of instruction is given to those who have passed this examination. Dr. Smith says: "The new regulation as to the use of mechanical restraint made by the Commissioners has rendered illegal the use of certain dresses which both my predecessor and myself had found to be of the greatest value in the treatment of some of the very acute cases admitted to the hospital." Nurse Susan Maskell received a pension after 22 years' service.

